# Groomed Fingerprint Sebum Sampling: Reproducibility and Variability According to Anatomical Collection Region and Biological Sex

**DOI:** 10.3390/molecules30030726

**Published:** 2025-02-06

**Authors:** Madeline Isom, Eden P. Go, Heather Desaire

**Affiliations:** Department of Chemistry, University of Kansas, Lawrence, KS 66045, USA; madeline.isom@ku.edu (M.I.); edenp@ku.edu (E.P.G.)

**Keywords:** sebum, mass spectrometry, fingerprint, biomarkers, lipidomics, lipids, machine learning, skin, noninvasive sampling

## Abstract

Sebum lipids, accessible via groomed latent fingerprints, may be a valuable, underappreciated sample source for future biomarker research. Sampling sebum lipids from the skin is painless for patients, efficient for researchers, and has already demonstrated the potential to contain disease biomarkers. However, before sebum sampling can be implemented in routine studies, more information is needed regarding sampling reproducibility and variability. This information will enable researchers to choose the best practices for sebum-based studies. Herein, we use our recently established workflow for the collection and analysis of groomed fingerprints to assess the reproducibility of lipid profiles obtained via mass spectrometry. Using 180 fingerprint samples collected from 30 participants, we also assess lipid changes according to biological sex and anatomical grooming region (cheek, neck, and forehead) via supervised and unsupervised classification. The results demonstrate that this sampling protocol achieves satisfactory reproducibility, and negligible differences exist between male and female groomed fingerprint lipids. Moreover, the anatomical grooming region can impact the fingerprint lipid profile: cheek- and forehead-groomed fingerprints are more similar to one another than either collection site is to neck-groomed fingerprints. This information will inform future sebum-based biomarker investigations, enabling researchers to collect meaningful lipidomic datasets from groomed fingerprint samples.

## 1. Introduction

Sebum, the oily mixture coating the entirety of the skin surface, may be an ideal, underexplored sample source for biomarker exploration studies. Sebum contains a variety of readily accessible lipids, including triglycerides, wax esters, fatty acids, cholesterol esters, cholesterol, and squalene [1,2,3,4]. In recent studies, changes in sebum lipids have been correlated to diseases such as Parkinson’s disease [5,6,7], COVID-19 [8,9], atopic dermatitis [10,11], and diabetes [12]. These preliminary findings suggest sebum lipids may be useful analytes for biomarker research. Furthermore, sampling sebum lipids from the skin surface is ideal for biomarker studies, as it does not cause discomfort to the patient and is entirely noninvasive compared to the traditional blood draw, the technique that is most commonly used [1,13]. Protected populations, such as children and elderly patients, who tend to be underrepresented in current research [14,15], might, therefore, be better represented if the requirement for sampling is a painless touch to the skin surface rather than a needle-in-arm blood draw. An added benefit, sampling from the skin surface allows for many samples to be collected rapidly, enabling the acquisition of large data sets that are compatible with powerful machine learning applications downstream. Since sebum lipids have already demonstrated preliminary diagnostic potential [5,6,7,8,9,10,11,12], and sample collection is painless and efficient, skin surface sebum sampling could be an advantageous approach to future biomarker investigation.

Though sebum sampling is becoming increasingly common among the metabolomics community [13], it is not yet widely used in biomarker research. Before specific applications of sebum sampling can be considered a viable alternative to mainstream, blood-based sampling protocols, more information is needed regarding sample reproducibility and variability so that researchers may design suitable experiments with this information in mind. For example, researchers need to know whether the MS data for lipids acquired from sebum sampling are sufficiently reproducible, compared to established methods, before this approach would be chosen over other methods for large scale lipidomic studies. Additionally, the inherent biological differences that exist among participants, such as that pertaining to participant demographics (ex. biological sex), could impact the sebum lipid profile [16,17,18], and this should be better understood. Moreover, since many preliminary studies utilize groomed fingerprint sebum sampling in which participants touch other areas of the body prior to depositing fingerprints [4,19,20,21,22,23,24,25], it will be important to identify the extent to which different anatomical grooming regions may impact the groomed fingerprint lipid profile so that experiments can be designed with this in mind. A better understanding of sebum sampling reproducibility and variability will enable researchers to make informed decisions about the collection procedure as well as the data processing steps that will yield the most meaningful data sets for biomarker exploration. Furthermore, understanding the inherent intragroup variation (i.e., among healthy patient controls) will allow researchers to design future biomarker studies that leverage this information in a way that maximizes intergroup variation (i.e., between healthy and diseased patients), thus unlocking the potential of sebum lipids as useful biomarkers of disease.

In prior studies, researchers have investigated the extent to which the donor’s sex impacts the sebum lipid profile, and results vary [1,26,27]. Some of the research suggests overall sebum content is higher in males than in females [11,28,29,30]. Specifically, it has been found that skin surface fatty acids and ceramides may vary according to donor sex [16,17]. In addition, one research group was able to classify male and female sebum samples with 89% accuracy using mass spectrometry (negative ion mode) and machine learning, indicating discriminatory lipidomic features exist between male and female participants [18]. However, other studies have contradicted these results and reported that no significant differences were observed between male and female sebum production [27,31,32,33]. While researchers have found age [34] and exercise [19] to differentially effect the skin surface lipid profiles of males and females, these same research groups also reported that the two sexes could not be effectively distinguished according to their skin surface lipids [19,34]. Likewise, one group that assessed the fatty acid profiles of groomed fingerprints found no significant differences between male and female samples [4]. Another group that investigated sebum triglycerides found that while two triglycerides showed significant differences between male and female samples, the majority of the triglycerides did not vary with donor sex [35]. With such conflicting reports, it is important for further research to assess whether biological sex effects the groomed fingerprint lipid profile, and if so to what extent, so that these differences can be accounted for in future sebum-based studies.

Another variable that may contribute to unwanted intragroup sample variability, and thus is worthy of further investigation, is the anatomical region of sebum collection, specifically for that of groomed fingerprint lipids. In many sebum sampling approaches, groomed fingerprints are collected, which require donors to briefly touch areas of the body that are high in sebum secretion prior to depositing latent fingerprints [4,19,20,21,22,23,24,25]. This grooming technique is often performed in order to enhance detection of the lipid profile [4,19,25]. In some cases, donors collectively touch multiple different anatomical regions prior to donating fingerprints [4,20,21,22,23,24]. However, other researchers have found sebum lipids to vary according to anatomical collection region [1,28,34,36]. Since different anatomical regions may produce different abundances of lipids, it is possible that differences in fingerprint grooming protocols could cause unwanted variability among groomed fingerprint samples. If this is the case, it could make results less transferable between research groups. Moreover, a greater variability among like samples (i.e., among control samples) can make it more challenging for machine learning algorithms to parse out the subtle differences between unlike samples (i.e., disease vs. control samples). In previous work, our pilot study of six participants suggested that grooming region may affect the lipid profile of groomed latent fingerprints [37], but because the sample set was small, more research using a larger participant pool is necessary before researchers can make fully informed decisions regarding best practices for groomed fingerprint collection.

Herein, we assess overall sample reproducibility as well as potential sources of variability present in groomed fingerprint sebum samples. Using the previously established workflow for the collection and analysis of groomed latent fingerprints [37], we evaluate the reproducibility of fingerprint lipid profiles collected from three anatomical grooming regions (neck, cheek, and forehead). In addition, we explore the extent to which participant sex and anatomical collection region impact the groomed fingerprint lipid profiles of 180 fingerprint samples collected from 30 participants. This information regarding groomed fingerprint sampling variability will enable researchers to choose best practices for groomed fingerprint sample collection. Using the results of this study, sebum-based biomarker experiments can be designed to account for potential intragroup variability so that intergroup variability can be readily observed. The information obtained herein will promote effective lipidomic studies that utilize noninvasive sebum sampling methods, an endeavor that will be especially useful for including protected populations in biomarker research and efficiently acquiring a large number of samples.

## 2. Results and Discussion

**Figure 1:**
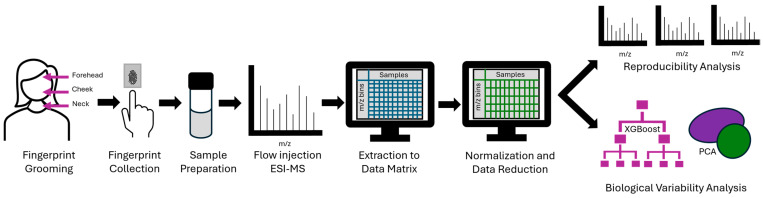
Schematic of the workflow used for groomed fingerprint collection and mass spectrometry data analysis.

The workflow used to collect and analyze groomed fingerprint samples is shown in Figure 1. Participants groomed their forehead, cheek, and neck regions and deposited their groomed fingerprints onto aluminum foil. Samples were prepared in organic solvent and desalted via liquid–liquid extraction. High-resolution MS data were acquired, and the raw data were extracted into a data matrix of samples and features. This matrix was normalized, and low-abundant peaks were removed. The MS data collected from groomed fingerprint samples was used to assess the MS sample reproducibility and determine the relative impact of two biological variables: anatomical grooming region and biological sex. This analysis was accomplished via supervised (XGBoost) and unsupervised (PCA) classification. These experimental results will be useful to researchers interested in leveraging the advantages of sebum sampling in a way that achieves the most meaningful lipidomic data sets for routine studies.

### 2.1. Reproducibility

The purpose of this experiment was to optimize and assess the reproducibility of the replicate, groomed fingerprint samples within a single MS batch as well as across multiple MS batches. To optimize sample reproducibility, low-abundant peaks were removed, and the data set was normalized in two different ways. First, *m*/*z* bins were excluded from the matrix unless at least 1% of the samples contained a nonzero number. This removes the bins that do not contain sufficient data. Matrix values were then normalized to reflect fractional portions of the total ion count of the corresponding sample, as described in the Section 3. This step adjusts for slight differences in the lipid concentrations (which are present because there is variability among the total lipid content from one sample to another). Next, 1 × 10^−10^ was added to each value in the matrix so that only non-zero values would be present, and log_2_ transformation was performed. This step reduces the influence of outliers on the data. Finally, samples were normalized according to MS batches using removeBatchEffect normalization. This step corrects for slight differences that occur due to the sample injection order or batch. After normalization, only *m*/*z* bins containing the top 10% of median intensity values were kept, resulting in a total of 3000 *m*/*z* bins in the final feature matrix.

**Figure 2:**
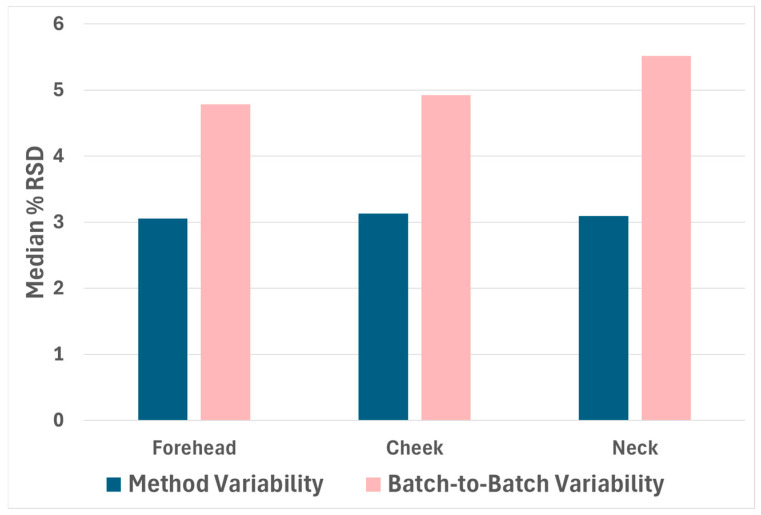
Intrabatch and interbatch reproducibility (median % RSD of peak intensities across *m*/*z* bins) of groomed fingerprint MS data. Data are representative of six technical replicates injected across five MS batches (grooming regions analyzed separately).

Figure 2 shows a bar graph depicting the median % RSD for each grooming region, as outlined in Section 3.5 of the methods section. For technical replicates, the median % RSD of peak intensities was approximately 3.1% for each grooming region, suggesting samples are highly reproducible within a single MS batch. For repeat injections across five MS batches, the median % RSD for the peak intensities was approximately 4.8%, 4.9%, and 5.5% for forehead, cheek, and neck grooming regions, respectively, demonstrating ideal batch-to-batch reproducibility as well. Overall, this experiment demonstrates that this method of sebum sampling and MS data processing results in highly reproducible data sets, both among replicate samples within a single MS batch as well as across consecutive MS batches. However, we note that across larger MS batches containing a high number of samples, the median % RSD is likely to increase, and MS batches injected on separate days may demonstrate greater variability.

### 2.2. Sex-Based Differences

If differences exist between male and female sebum lipids, then researchers will need to be aware of these differences when designing sebum-based biomarker studies. The purpose of this portion of the study was to identify the extent to which biological sex impacts the groomed fingerprint lipid profile. Via supervised and unsupervised classification, sex-based differences were assessed using the lipid profiles of 180 samples collected from 30 participants.

**Figure 3:**
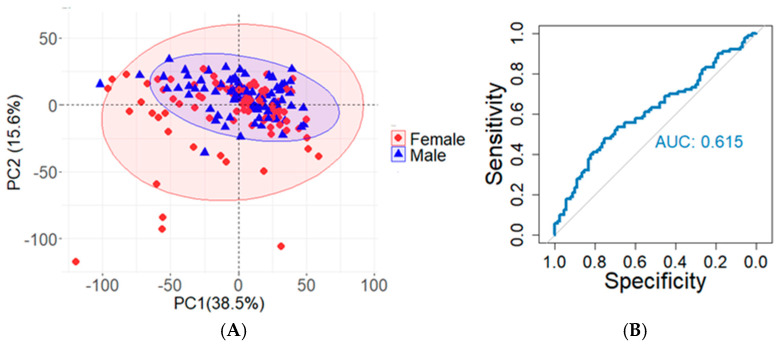
Unsupervised and supervised classification results for 90 male and 90 female samples collected from 30 participants. (**A**) Principal component analysis (PCA) of 180 groomed fingerprint samples; concentration ellipses are generated with RStudio using packages “factoextra” and “ggplot2”. (**B**) ROC curve reflecting the classification results for the same 180 groomed fingerprint samples from panel A. The AUC is 0.615, and 57% of the samples are correctly classified.

Figure 3A shows the PCA plot of 90 male and 90 female samples, where each point represents a sample, and the two axes represent the linear combination of maximum variability. The PCA plot shows overlap between male and female sample groups, suggesting there are not clear discernable differences between the detected lipids of male and female donors. Using the same samples for XGBoost classification yields a similar outcome, as only 57% of the samples are correctly classified, and the AUC value for the ROC curve (shown in Figure 3B) is 0.615. Based on the results, male and female samples do not appear to be measurably different among these 30 donors. Others have found similar results [19,27,31,32,33,34]. Furthermore, when unnormalized MS data are used to reclassify the same 180 sebum samples, results continue to depict a lack in discernable differences related to participant sex, as shown in the Supporting Information (Figure S1). This information is useful, as it suggests that including both males and females in biomarker studies is not likely to introduce the extra variability researchers need to account for.

### 2.3. Anatomical Region Variability

**Figure 4:**
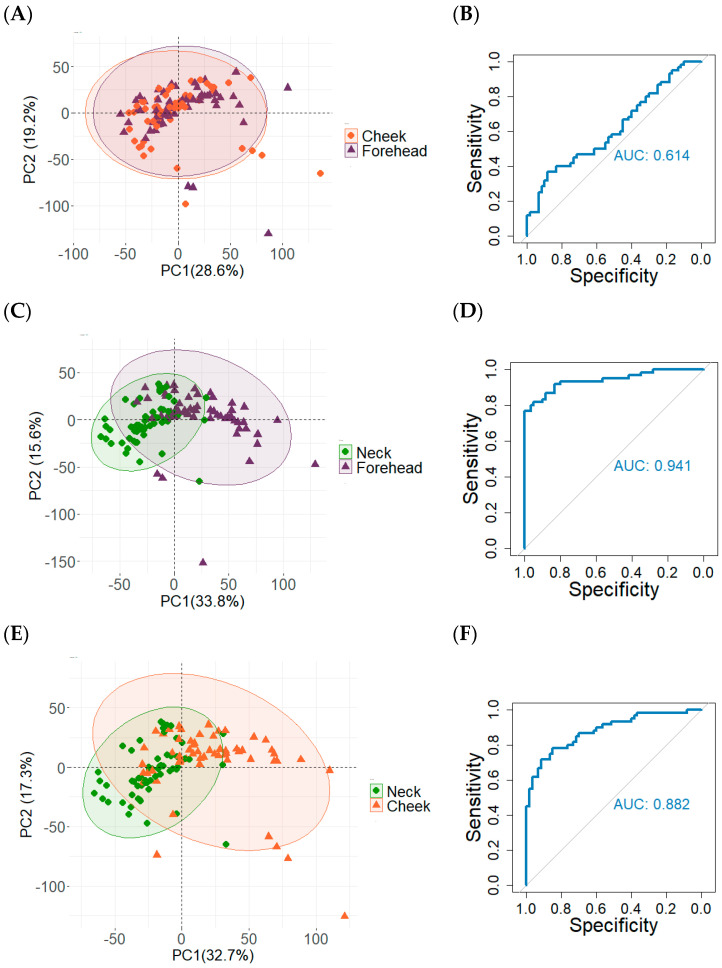
Unsupervised and supervised classification results for 180 samples collected from 30 participants using three anatomical grooming regions. Principal component analysis (PCA) concentration ellipses show sample distribution and overlap, generated with RStudio using packages “ggplot2” and “factoextra”. (**A**) PCA of 120 total cheek- and forehead-groomed fingerprint samples collected from 30 donors. (**B**) ROC curve reflecting the classification results for the same 120 cheek- and forehead-groomed samples from panel (**A**). The AUC is 0.614, and 54% of the samples are correctly classified. (**C**) PCA of 120 total neck- and forehead-groomed fingerprint samples collected from 30 donors. (**D**) ROC curve reflecting the classification results for the same 120 neck- and forehead-groomed samples from panel (**C**). The AUC is 0.941, and 86% of the samples are correctly classified. (**E**) PCA of 120 total neck- and cheek-groomed fingerprint samples collected from 30 donors. (**F**) ROC curve reflecting the classification results for the same 120 neck- and cheek-groomed samples from panel (**E**). The AUC is 0.882, and 81% of the samples are correctly classified.

Since different anatomical regions can contain different lipids and overall abundances [28,34,36], it is possible that the grooming location may impact the resulting fingerprint lipid profile and, therefore, would be an important consideration for the sebum-sampling study design. Thus, the goal of this portion of the study was to identify whether different anatomical grooming regions result in measurable differences among groomed fingerprint lipid profiles. Figure 4 shows the PCA and classification results using XGBoost for each grooming region comparison (120 samples for each analysis). Cheek–forehead classification results demonstrate considerable group overlap (PCA, Figure 4A) and poor discrimination during supervised classification (54% accuracy, AUC = 0.614, Figure 4B), suggesting cheek- and forehead-groomed fingerprint lipid profiles are not readily discernable from one another. In contrast, neck–forehead classification results show noticeable separation of data clusters (PCA, Figure 4C) and a more accurate supervised classification outcome (86% accuracy, AUC = 0.941, Figure 4D). Similarly, neck–cheek comparisons also show separation of sample sets (PCA, Figure 4E) and effective discrimination by supervised classification (81% accuracy, AUC = 0.882, Figure 4F). Together, these results suggest that neck-groomed fingerprint samples are measurably different from cheek- and forehead-groomed fingerprint samples.

These results are similar to those obtained in our previous proof-of-concept study on a smaller sample set collected from six individuals [37], as well as results obtained in other experiments using sebumeter measurements [36]. Since anatomical grooming region can impact the fingerprint lipid profile (in the case of the neck-groomed samples), researchers would benefit from choosing a single grooming region rather than collectively grooming different regions of the body, as this technique could introduce unnecessary variability into the data set and make results less transferable across different research groups.

## 3. Materials and Methods

### 3.1. Sample Collection

A single female participant, age 25, donated fingerprint samples for the reproducibility analysis. To assess fingerprint variability regarding participant sex and anatomical grooming region, 30 participants donated fingerprint samples. Participants ranged between 18 and 40 years old, included multiple ethnicities, and consisted of 15 males and 15 females. Groomed fingerprint samples were collected according to our previously described protocol [37] in compliance with The University of Kansas’ Human Research Protection Program for human subjects. Participants gave informed consent, wiped their hands with a hand sanitizing wipe, and then allowed their hands to air dry. Once their hands were dry, each participant rubbed their hands together for 10 s and then rubbed two fingertips to each of the three anatomical regions of study (forehead, cheek, and neck) for five seconds. After five seconds of grooming, participants placed each of their fingertips for 10 s onto clean aluminum foil sheets (approximately 1 cm × 2 cm each). For the reproducibility analysis, the participant used six fingers (index, middle, and ring fingers of both hands) to groom a single anatomical region, resulting in six replicate samples. This procedure was repeated for each of the three grooming regions, wiping the hands with a new sanitizing wipe and allowing them to air dry between each grooming protocol, resulting in a total of 18 samples. For the remaining analyses, each of the 30 participants used both index fingers to groom the forehead, middle fingers to groom the cheeks, and ring fingers to groom the back of the neck. This procedure resulted in 6 fingerprint samples per participant, and a total of 180 fingerprint samples among the 30 participants.

### 3.2. Sample Preparation

Immediately after fingerprint deposition, participants removed their fingers, and the foil was folded in half and placed into a glass vial with 400 µL of dichloromethane. The samples were sonicated for 10 min. The foil was then discarded, and 200 µL of distilled water was added to the vial to allow phase separation. The samples were sonicated again for 10 min and then left at room temperature for approximately 20 min to ensure adequate phase separation. The aqueous layer was removed, and the remaining organic layer was stored at −20 °C until MS analysis. Prior to MS analysis, samples were removed from the freezer and equilibrated to room temperature. Samples were then vortexed and further diluted by a factor of 20.

### 3.3. Flow Injection ESI-MS

High resolution flow injection electrospray ionization mass spectrometry was performed using a Waters Acquity UPLC instrument (Milford, MA, USA) coupled to an Orbitrap Fusion Tribrid mass spectrometer (Thermo Fisher Scientific, San Jose, CA, USA). Prior to ESI-MS analysis, samples were randomized according to the variable of study. For the reproducibility analysis, replicate samples of the three grooming regions were randomized, and the randomized series of 18 samples was injected a total of five times to produce five MS batches. For the analysis of grooming region differences among 30 participants, samples were injected in order of donor sex but randomized according to grooming region (forehead-, cheek-, and neck-groomed). For the analysis of sex-based differences among 30 participants, samples were injected in order of grooming region but randomized in reference to donor sex.

All MS data were acquired in the positive ion mode across a scan range of 250–1300 *m*/*z*. Specific flow injection and MS parameters followed a previous protocol [37]. Briefly, 5 μL of sample from the autosampler were directly injected into the mass spectrometer at a flow rate of 25 μL/min. An isocratic elution was employed using 50% Mobile Phase A for a 2 min duration. Mobile Phase A consisted of 50:50 methanol/water, and Mobile Phase B consisted of 20:79:1 acetone/2-propanol/water, both containing 5 mM ammonium acetate. One full scan was acquired every three seconds in the Orbitrap with MS maximum injection time, resolution, and AGC at 100 ms, 120,000 at 200 *m*/*z*, and 4 × 10^5^, respectively. Sheath gas, auxiliary gas, spray voltage, and capillary temperature were each maintained at 10 units, 6 units, 2.8 kV, and 275 °C, respectively.

### 3.4. Data Processing

Feature matrices were constructed from the raw MS data and used for subsequent supervised classification, with XGBoost as the classifier [38], and unsupervised classification, using PCA, in a similar manner as described previously [37]. Briefly, the raw MS spectra were converted to MS1 format using Raw Convertor (version 1.2.0.1) [39], and an in-house matrix building script was used to read in each MS1 file and create each feature matrix. The script was written and processed in R, version 4.3.3 [40]. To build the feature set for the matrix, each mass spectrum (ranging from 250 to 1300 *m*/*z*) was divided into *m*/*z* “bins” of 0.01 Da width, and the peak intensities within each bin were summed. This process was carried out for each sample across the 0.8–1.0 min retention time frame. The summed peak intensities were input into the matrix so that each row represented an *m*/*z* bin, and each column represented a sample. This resulting feature matrix, with corresponding features (*m*/*z* bins) and samples, allowed the data to be applicable for machine learning applications.

After the matrix was constructed, only the *m*/*z* bins in which at least 1% of the samples contained a nonzero value were kept. This procedure allowed for the exclusion of unused bins and decreased the total number of features in the matrix. Samples were then normalized so that each value in the matrix represented a fractional portion of the corresponding sample’s total ion counts. To accomplish this, each of the values within the feature matrix were divided by the sum of intensities from the corresponding column (sample). This normalization was employed to account for the fact that some individuals might produce a higher concentration of lipids than other individuals. Following sample normalization, 1 × 10^−10^ was added to each value in the matrix to eliminate zero values; the matrix was log_2_ transformed, and batch normalization was performed using removeBatchEffect via the R package “limma” [41] as this approach proved to be useful in similar sample sets [42]. To generate the removeBatchEffect vectors corresponding to sample injection order, a five-variable batch vector was used in the reproducibility analysis, and a six-variable batch vector was used in each of the variability analyses. For the six-variable vector, 30 consecutive samples were considered a single MS batch for a total of six batches (180 samples total). To minimize the noise in each feature matrix, the *m*/*z* bins with approximately the top 10% of median values were kept for subsequent XGBoost and PCA. For the reproducibility and sex-based analyses, 3000 *m*/*z* bins were included in the final feature matrices. For the analysis of grooming regions, 4000 *m*/*z* bins were included in the final feature matrices. The raw feature matrices are shown in Table S1, and the normalized feature matrices are shown in Table S2 of the Supporting Information. Feature matrices were transposed prior to machine learning applications.

### 3.5. Data Analysis: Sample Reproducibility

To evaluate the MS sample’s interbatch and intrabatch reproducibility, new data sets were acquired that contained 30 MS spectra (six technical replicates, each injected five times into different MS batches) for each of the three grooming regions. Reproducibility was assessed according to the percent relative standard deviation (% RSD) of the peak intensity for each *m*/*z* bin across replicate samples. To evaluate the sample reproducibility within a single MS batch (the method variability), the % RSD was calculated for the six technical replicates within each MS batch independently (*N* = 5). To assess the batch-to-batch reproducibility, the % RSD was calculated for the five repeat injections of each sample independently (*N* = 6). In both cases, the median % RSD value was used as the metric for evaluating reproducibility. This analysis was carried out for each anatomical grooming region (neck, cheek, and forehead) separately.

### 3.6. Data Analysis: Sample Variability

The feature matrices resulting from the 180 samples collected from 30 participants were analyzed via XGBoost (extreme gradient boosting) and PCA (principal component analysis). Classifications according to biological sex and anatomical grooming region (forehead, cheek, and neck) were performed. The package “xgboost” was used for supervised classification via XGBoost, and the packages “factoextra” [43] and “ggplot2” [44] were used for visualizing the results of unsupervised classification via PCA. For XGBoost classification, hyperparameters were not optimized. They included: the booster (gbtree), objective (binary:logistic), eta (0.3), gamma (0), max_depth (6), min_child_weight (1), subsample (1), colsample_bytree (1), and nrounds (50). In each classification, a variation in leave-one-out cross-validation (LOOCV) was employed so that samples were excluded from the training set if they originated from the same person as the sample being tested. This procedure ensured that the variable of study (ex. biological sex or anatomical region) was assessed authentically, and that there was no donor bias in the classification. Note: while a separate test set was not part of the study design, the hyperparameters of the classifier were not optimized in any way, so the LOOCV results ideally approximate the true accuracy obtainable by an independent test set, had more human participants been available for sampling [45]. Classification accuracy was measured according to the percentage of correctly classified samples, and AUC values were calculated from the ROC curves produced via XGBoost classification using the package “proc” [46].

## 4. Conclusions

The results reported here inform biomarker exploration studies that employ sebum sampling techniques. Since sebum sampling is entirely painless and noninvasive, this approach is ideal for protected populations. Additionally, since many samples can be collected quickly, this sampling method is inherently useful for machine learning applications, of which large numbers of samples are valuable for improving performance. Herein, we have demonstrated exceptional reproducibility for MS data collected from groomed fingerprint sampling, as well as identified the extent to which the biological sex of the donor and the anatomical grooming region will measurably impact the resulting fingerprint lipid profile. This method of fingerprint lipid collection and analysis demonstrates excellent reproducibility both within a single MS batch and across multiple MS batches. Donor sex does not appear to substantially impact the groomed fingerprint lipid profile among the donors tested. Anatomical collection region used in the grooming protocol can impact the resulting fingerprint lipid profile, and these results indicate that cheek- and forehead-groomed fingerprint samples are more similar than those acquired by neck-grooming. These experiments introduce important findings that will be necessary for obtaining meaningful data sets in biomarker studies. In the future, sebum sampling may be useful in distinguishing between healthy and disease patient groups, and the outcomes presented herein will aid in this important endeavor.

## Data Availability

All raw data can be found in Table S1 of the Supplementary Materials.

## Supplementary Materials

The following supporting information can be downloaded at: https://www.mdpi.com/article/10.3390/molecules30030726/s1, Table S1: Raw Feature Matrices; Table S2: Normalized Feature Matrices; Figure S1: Male/Female Classification using Unnormalized MS Data.
