# Supplementary material for: Groomed Fingerprint Sebum Sampling: Reproducibility and Variability According to Anatomical Collection Region and Biological Sex
Source: Molecules. 2025 Feb 6;30(3):726. doi: 10.3390/molecules30030726 (PMC11820395; doi:10.3390/molecules30030726)
Supplement: Supplementary file 1 [file molecules-30-00726-s001.zip › Molecules_SI_Jan2025.pdf]

Supporting Information for:

Groomed Fingerprint Sebum Sampling: Reproducibility and Variability According to Anatomical Collection Region and Biological Sex

Madeline Isom, Eden P. Go and Heather Desaire *

Department of Chemistry, University of Kansas, Lawrence, KS 66045, USA;
madeline.isom@ku.edu (M.I.); edenp@ku.edu (E.P.G.)
* Correspondence: hdesaire@ku.edu; Tel.: +1-785-864-3015

Table of Contents:

Cover page	S1
Table S1	(xlsx file)
Table S2	(xlsx file)
Figure S1	S2

A

B

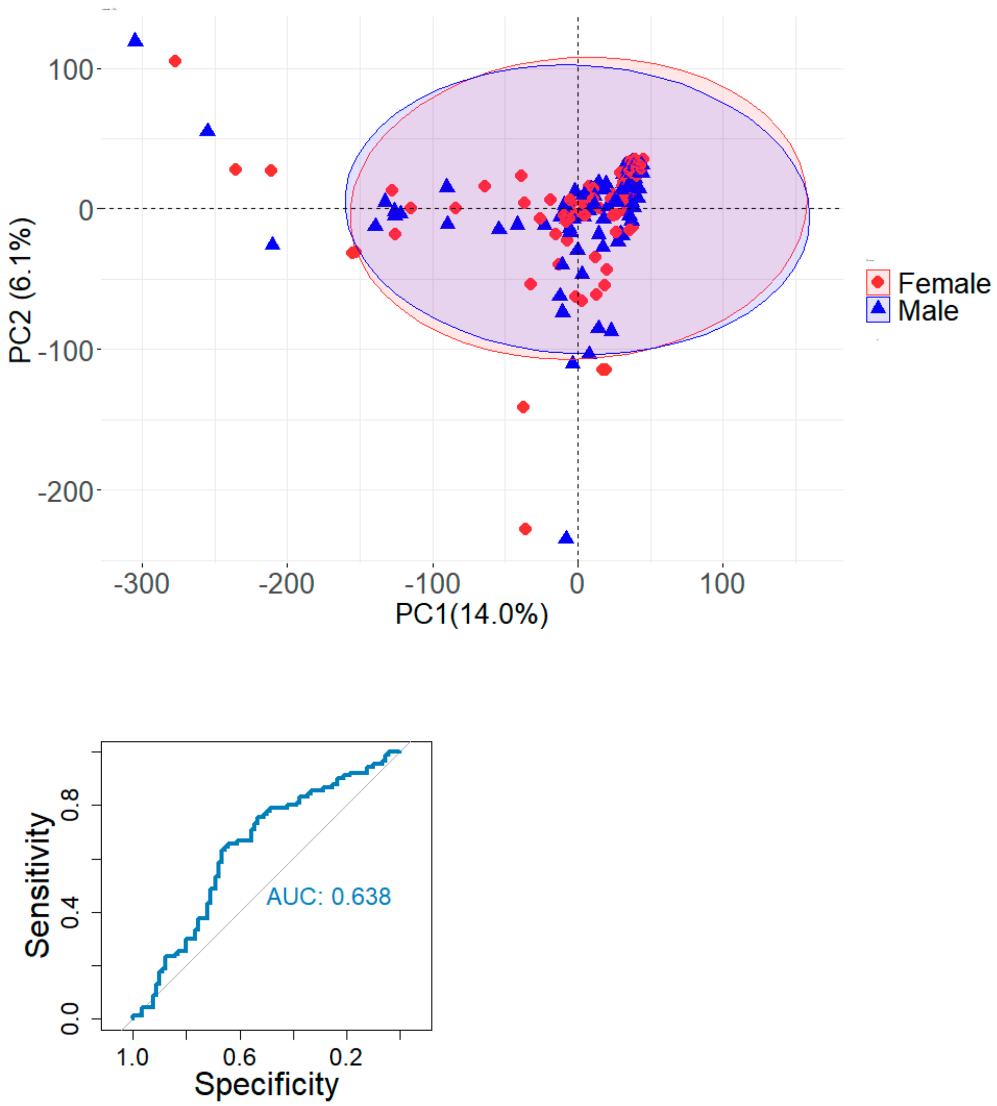

Figure S1. Unsupervised and supervised classification results for 90 male and 90 female samples collected from 30 participants. Results are representative of unnormalized MS data. (A) Principal component analysis (PCA) of 180 groomed fingerprint samples; concentration ellipses are generated with RStudio using packages “factoextra” and “ggplot2.” (B) ROC curve reflecting the classification results for the same 180 groomed fingerprint samples from panel A. The AUC is 0.638, and 63% of the samples are correctly classified.
